# Macromastia: an economic burden? A disease cost analysis based on real-world data in Germany

**DOI:** 10.1007/s00404-020-05841-7

**Published:** 2020-10-29

**Authors:** Sebastian M. Jud, Anne Brendle-Behnisch, Carolin C. Hack, Caroline Preuss, Andreas Arkudas, Raymund E. Horch, Matthias W. Beckmann, Michael P. Lux

**Affiliations:** 1 Department of Gynecology and Obstetrics, Erlangen University Hospital, Friedrich Alexander University of Erlangen–Nuremberg, Erlangen, Germany; 2 Department of Plastic Surgery and Hand Surgery, Erlangen University Hospital, Friedrich Alexander University of Erlangen–Nuremberg, Erlangen, Germany; 3 Department of Gynecology and Obstetrics, Frauenklinik St. Louise, Paderborn, St. Josefs-Krankenhaus, Salzkotten, Frauen- Und Kinderklinik St. Louise, St. Vincenz GmbH, Husener Strasse 81, 33098 Paderborn, Germany

**Keywords:** Macromastia, Gigantomastia, Disease costs, Conservative treatment, Health care, Breast reduction mammoplasty

## Abstract

**Purpose:**

Symptomatic macromastia causes physical and psychological problems that can lead to restrictions in the patients’ social and working lives and a reduced quality of life. Associated medical treatments also have a considerable impact on health-care costs. Several studies have assessed these costs, but the total disease costs of macromastia have never been evaluated on the basis of real-world data.

**Methods:**

The data for 76 patients who underwent reduction mammoplasty between 2008 and 2016 were collected using a two-part questionnaire (preoperative and postoperative), as well as the patient files. Topics surveyed, besides demographic data, included physician visits, medical imaging, medical procedures, medical treatments, rehabilitation and convalescent measures, drug intake, medical aids, exercise activity, and sick leave days before surgery, to calculate the costs per year of conservative treatment of symptomatic macromastia.

**Results:**

The mean time from start of symptoms to surgery was 11.82 years. The data for this group of patients with symptomatic macromastia show that costs per patient amount to €1677.55 per year. These costs include medical consultation, radiological imaging, medical treatments and procedures, physical therapy and rehabilitation, medication, special brassieres, exercise classes costs for sick leave due to problems with macromastia, and travel expenses.

**Conclusions:**

These results show that considerable health-care costs arise due to macromastia with conservative treatment. Overall, macromastia costs €1677.55 per patient/year. In particular, lost productivity due to sick days and the costs of physiotherapy are factors driving the high costs.

## Introduction

There is no unique definition of macromastia. There are several different synonyms for it, such as gigantomastia and hyperplasia of the mammary gland. Macromastia is usually defined as excessive growth of the mammary gland beyond the normal range [1]. However, what is normal always lies in the eye of the beholder and may vary between countries and cultures, with fluid transitions along a continuum. Another definition is based on the symptoms: chronic pain in at least three anatomic regions, triggered by large breasts [2].

The precise cause of macromastia is still as yet unknown. Several triggers or causes have been reported, such as hormone-related causes, anovulation, and mutations in oncogenes [3–6]. As the definitions used are not standardized, there are no exact figures for the incidence of the condition, but it is thought that 1–5% of the female population suffer from macromastia [4].

Breast hypertrophy or macromastia in patients is often accompanied by several somatic problems such as chronic pain in the chest, shoulders, back, and neck, degenerative changes in the spine, and headaches [2, 7–20]. Neurological problems such as dysesthesia and carpal tunnel syndrome have been reported in the literature [11, 14, 21, 22]. Patients often wear special brassieres to support the breast and obtain some relief in the shoulder and neck area. This results in strap grooves, which may in some cases be irreversible and painful. Due to the skin contact between the breast and the abdomen/thorax, it is easy for irritations of the skin, eczema, and intertrigo to develop. Shortness of breath has even been reported [2, 8, 10–12, 14, 16–19, 23, 24].

Due to the chronic pain, patients often reduce their leisure activities [9, 11, 16, 25]. This reduction of social and cultural interactions leads to body perception disorders and reduced self-confidence, and can even cause depression [16, 19, 26, 27]. These are all also reasons for reduced productivity and inability to work.

Although the most effective and normally the only causal treatment is reduction mammoplasty, this type of surgery is often regarded as cosmetic surgery by patients, insurance companies, and even by medical professionals. In addition to weight reduction, conservative treatments are, therefore, often recommended by insurance companies, with approval for insurance coverage of surgical costs depending on previous implementation of such treatments. Treatments that are often recommended include physiotherapy, fango, massage, and acupuncture. Insurance companies may recommend nutritional counseling to promote weight loss, or psychotherapy to strengthen body perception [7, 28]. Special brassieres are intended to provide support for the skeletal system, and in combination with strengthening of the musculoskeletal system may lead to pain relief. In many cases, pain relief is achieved by medication alone. Insurance companies sometimes advise the patient to undergo rehabilitation measures [11, 14, 19, 20, 28]. However, none of these conservative treatments has shown sufficient and long-lasting effects, as they do not represent causal therapies [11, 29].

All of these treatments create costs. Several studies have compared the costs of conservative treatment with surgical procedures, but, to the best of our knowledge, there has been no research analyzing the precise costs arising for insurance companies and in particular for society as a whole. More detailed information about these costs may be helpful in health-care decision-making. The aim of the present study was, therefore, to fully calculate the costs arising for insurance companies, and society for a patient with macromastia.

## Materials and methods

### Study design and patient recruitment

This is a retrospective study including patients who underwent surgery at the University Breast Center for Franconia (Departments of Gynecology and Plastic Surgery) from 2008 to 2016. The patients were identified from the hospital information system using the operation and procedure (OPS) codes 5–884 ff. (reduction mammoplasty) and ICD-10 diagnosis N62 (macromastia, mammary hypertrophy, mammary hyperplasia, and gigantomastia).

**Fig. 1:**
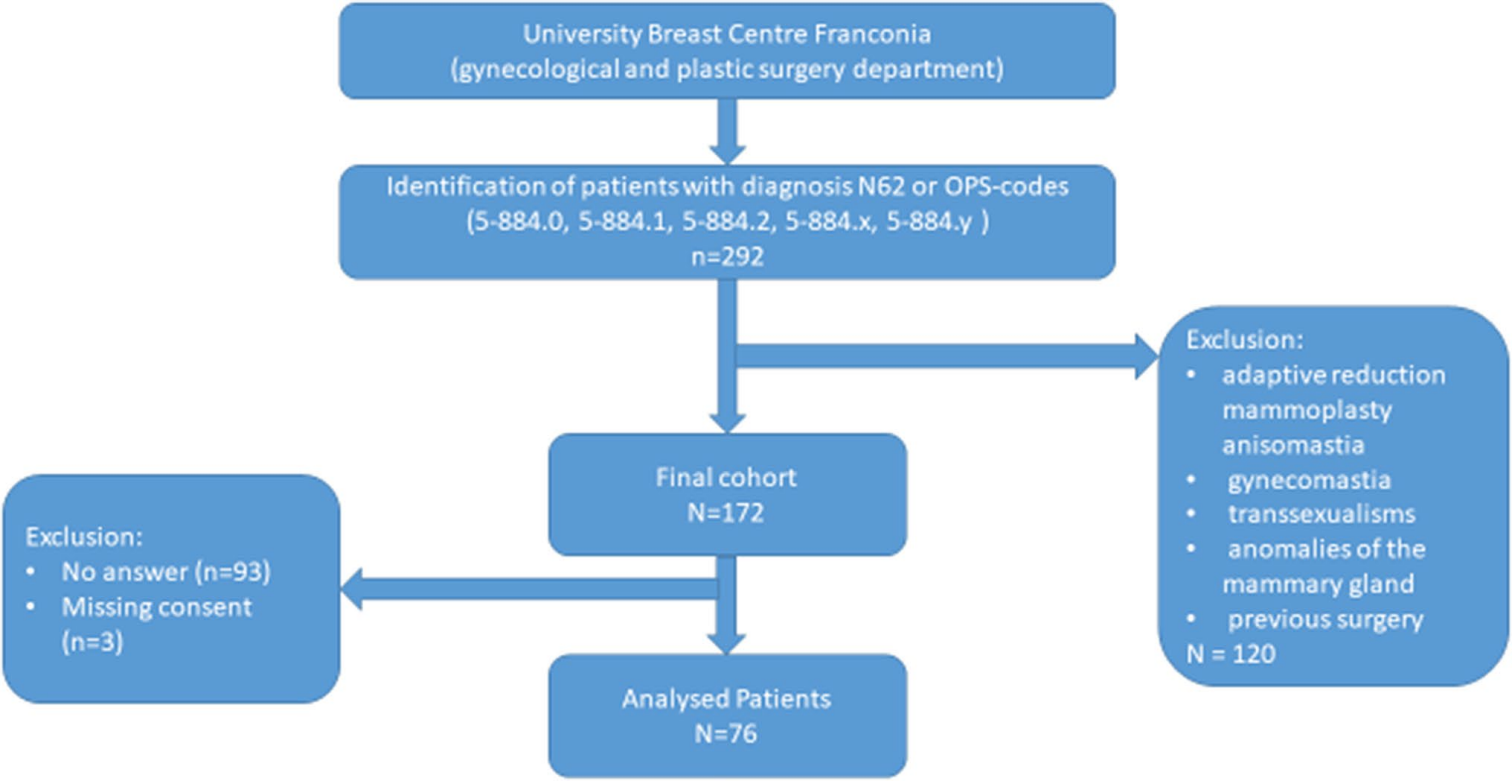
Patient selection

Patients with breast cancer, adaptive one-sided reduction mammoplasty, anisomastia, gynecomastia, transsexualism, anomalies of the mammary gland, and previous surgery were excluded (Fig. 1).

### Patients’ questionnaire

All of the patients were asked to complete a two-part questionnaire. In the first part, they were asked to report on the period before surgery. Part one comprised questions regarding:

- Patient’s characteristics.
- Medical consultations.
- Medical imaging procedures.
- Treatment procedures carried out by physicians.
- Medical treatments such as medical exercise, rehabilitation, and sports.
- Medication (including pain relief).
- Work status and sick days per year.

Information was also requested about reimbursement for these treatments by insurance companies and the availability and costs of objection procedures (when reimbursement is declined). The final item was about quality of life before surgery. All these items targeted known and often-recommended conservative treatments for symptomatic macromastia. The second part of the questionnaire was concerned with the postoperative period, including medical treatments as above, and also quality of life. The present study investigated part one as a complete, real-world disease cost analysis. Approval for the study was provided by the local ethics committee (ref. no. 34_16B). All data were anonymized. Data from the patients’ charts regarding surgery, pathology, and hospital stays were also retrieved.

### Cost accounting

Costs were analyzed per patient and year. The patients were either asked about numbers of procedures per year, from which the annual costs were calculated (e.g., for medical consultations); or the patients reported the total number of treatments during the period before surgery and the annual costs were calculated by dividing the total by the mean period between the onset of symptomatic disease and the time of surgery (e.g., for medication or imaging procedures). The figures were averaged for the complete cohort to calculate the mean frequency.

### Medical consultations

Patients were asked if they had consulted their family physician due to symptoms of macromastia. The number of consultations per year, numbers of nonproductive work days, travel expenses, and the duration of the consultations were recorded. This complex of questions was repeated for orthopedics, dermatology, neurology, gynecology, plastic surgery, psychiatry, psychotherapy, and psychology. For consultations with psychotherapists, the type of therapy (talking therapy, behavioral therapy, or hypnosis) had to be specified.

The basis used for cost accounting in the out-patient setting was the German 2015 Physicians’ Fee Schedule (*Einheitlicher Bewertungsmassstab,* EBM) [30]. Reimbursement was standardized to quarterly periods, and the basic flat rate was, therefore, calculated for a maximum of four times per year. If the consultation process lasted longer, an additional fee was calculated. Total annual costs represented the base rate plus consultation fees. Laboratory costs were not calculated. Different costs were calculated for each specialty in the same way (Table 1).

**Table 1:** General costs of procedures in different categories: medical consultations, imaging, medical procedures, medical treatments, educational courses, physical therapy, and rehabilitation in various specialties (Costs for medical consultation, imaging, and medical procedures are based on EBM, for medical treatment on the German Social Security Code.)

Category	Item	Cost/session or cost/unit (€)
Medical consultations	Family physician, base rate	12.53
	Medical consultation due to severity of the disease	9.24/10 min
	Orthopedics, base rate	18.69
	Dermatology, base rate	14.38
	Neurology, base rate	23.42
	Neurological consultation, neurologic treatment and diagnosis	9.24/10 min
	Gynecology, base rate	14.89
	Psychiatry, base rate	20.13
	Psychiatric consultation, psychiatric treatment and diagnosis	13.97/10 min
	Psychotherapy, base rate	12.33
	Probatory psychotherapy session	63.79
Medical imaging	Computed tomography	74.37
	Magnetic resonance imaging	124.60
	Conventional X-ray	12.69
	Ultrasound	12.54
Medical procedures	Acupuncture, base rate	48.28
	Acupuncture, treatment session	21.78
	Manual therapy of the spine	7.29
	Local anesthesia/neural therapy	8.04
	Dorn back therapy	21.00
Medical treatment	Physiotherapy	15.85
	Massage	11.52
	Fango	22.22
	Manual therapy	18.62
	Osteopathy	66.00
Special educational courses	Back therapy training	10.22
	Nutritional courses	58.80
Physical therapy	Electrotherapy	4.39
	Thermotherapy	3.05
	Heliotherapy	3.00
Rehabilitation and health resort treatment	Orthopedic rehabilitation (in-patient)	2752
	Orthopedic rehabilitation (out-patient)	1786
	Psychosomatic rehabilitation	6468
	Preventive treatment at a health resort	3013

Consultations at university hospitals were calculated on the basis of the 2015 university out-patient flat rate (*Hochschulambulanzpauschale*) for gynecology and plastic surgery in Bavaria (€103.30).

### Medical imaging

Patients were asked about medical imaging procedures that had been performed and their frequency in relation to macromastia symptoms. The patients had to state the type of imaging (e.g., CT, MRI, X-ray, and ultrasound) and the frequency. Cost accounting was carried out on the basis of the 2015 EBM. The mean costs of the examinations were calculated. Preoperative imaging was not calculated, as this is covered by the disease-related group for in-patient treatment and is not associated with conservative treatment. The various costs for medical imaging are listed in Table 1.

### Medical procedures

The patients were asked to list medical procedures they had undergone and their frequency in relation to macromastia. The patients reported acupuncture, chiropractic, local anesthesia, and osteopathy. These costs were calculated on the basis of the 2015 EBM and are presented in Table 1.

### Medical treatment

The patients were asked to provide information about the different medical treatments they had received due to macromastia and to report their frequency. They were asked about physiotherapy, massage, fango, osteopathy, and special educational courses for back therapy or nutrition. The costs for medical modalities such as massage, fango, and physiotherapy were calculated on the basis of §125 of the German Social Security Code (*Sozialgesetzbuch* V) using the Bavarian statutory insurance company (AOK, Allgemeine Ortskrankenkassen) as a reference point.

Costs for osteopathy were mainly not covered by insurance. The mean costs for osteopathic treatment were taken as indicated by the Professional Association of German Osteopathic Medical Associations (*Berufsverband Deutscher Osteopathischer Ärzteverbände*, BDOÄ) and Association of Osteopaths in Germany (*Verband der Osteopathen Deutschland e.V.*, VOD) [31, 32] (Table 1).

### Special educational courses

Costs for back therapy training, nutritional courses, or other educational courses were calculated by taking the standard costs from several institutions in Bavaria, calculated as costs per minute, and adjusting for inflation. Table 1 lists the costs for back therapy training and nutritional education courses.

### Physical therapy

Patients were asked about balneotherapy, electrotherapy, thermal therapy, hydrotherapy, and heliotherapy that had been performed and the frequency of such treatments, as described above. Costs were calculated in the same way as for medical treatment, based on the AOK Bavaria insurance company as a reference point (Table 1).

### Rehabilitation and treatment in health resorts

In addition, patients were asked about rehabilitation stays and treatments at health resorts associated with the symptoms of macrosomia. Frequencies and types were recorded, as well as travel expenses. The costs for these procedures were calculated on the basis of data from the German Pension Insurance Fund (*Deutsche Rentenversicherung*) [33]. Costs for preventive treatments were calculated with data from Federal Health Reports (*Gesundheitsberichterstattung des Bundes*) [34, 35].

### Medication

Patients were asked about medication due to symptoms related to macromastia. The various substances used were divided into specific groups: analgesics, antidepressants/psychopharmaceuticals, relaxants, ointments/dermatologic medication, and other medication. The patients had to provide information about the drug name, dosage, frequency, and duration.

Costs were calculated using drug prices from the German pharmacopeia, *Rote Liste 2015* (www.rote-liste.de). The least expensive drug and package size were always used. All prices were adjusted for inflation.

Frequency was classified into six categories (ranging from: twice daily—once daily—several times per week—once per week—several times per month—to once per month). Days of medication intake were calculated for each drug. The total was multiplied by the daily drug costs for each drug. Costs for all the drugs in the above categories were calculated.

### Special brassiere

Patients were asked if they had a special brassiere due to macromastia. The number of brassieres was reported. Patients were asked to state whether the brassiere was covered by insurance. Costs for the special brassieres were obtained from medical supply stores and were €70 on average. If they were covered by insurance, the patient had to pay 10% of the price.

### Physical exercise

Patients were asked if they had attended a fitness center or gymnastics club due to the symptoms of macromastia. The costs for these activities were reported and monthly costs calculated.

### Employment

Patients were asked about their employment before reduction mammoplasty, stating whether they had missed work due to symptoms of macromastia. The number of working hours lost was reported, as well as the number of sick days. To calculate costs due to absence from work, the gross domestic product (GDP) per gainfully employed person in Germany in 2015 was used, divided by average working days minus 24 days’ vacation [36]. The GDP was €309.95 per day.

### Statistics

Microsoft Excel was used for data collection. Data analysis was performed using IBM SPSS Statistics for Windows, version 24.0 (IBM Corporation, Armonk, New York). Normally distributed data are described as means and categorical data as percentage frequencies.

## Results

A total of 172 patients were identified at the first step. Seventy-six out of 172 questionnaires were returned completed (44.1%, *n* = 93 no answer; *n* = 3 missing consent). The patients’ mean age at the start of macromastia symptoms was 26.48 years, and their mean body mass index (BMI) was 27.93 kg/m^2^. The mean period between the start of symptoms and surgery was 11.82 years.

### Direct medical costs

#### Physician visits and care

Contact with the family physician due to symptoms of macromastia was reported by 55.3% of the patients. A total of 1.74 visits per year were calculated, with a mean duration of 8.07 min, resulting in a €21.80 base rate and €12.97 consultation fee per year, representing annual costs of €34.77.

The corresponding figures for orthopedists were 2.14 visits per year with a mean duration of 11.97 min, resulting in costs of €40.00 per year and patient. Figures for other specialities are listed in Table 2.

**Table 2:** Costs arising for visits to physicians in different specialities

Speciality	Contact reported (%)	Visits per year	Duration of consultation (min)	Annual cost (€)
General practitioner	53.3	1.74	8.07	34.77
Orthopedics	68.4	2.14	11.97	40.00
Dermatologist	22.4	0.39	2.67	5.61
Neurologist	18.4	0.42	5.10	11.82
Gynecologist^a^	75.0	1.46	14.75	21.74
Plastic surgeon^a^	60.5	1.09	15.39	112.60
Psychologist	11.8	0.37	3.99	6.44
Psychiatrist	6.6	0.06	1.46	1.33
Psychotherapist	13.2	1.10	4.86	20.38

^a^Costs for university out-patient departments were calculated and added

#### Imaging

Almost two-thirds (73.3%) of the patients had undergone imaging procedures due to symptoms of macromastia. The costs for imaging procedures were calculated by multiplying the number of procedures with the above-calculated prices. Costs for computed tomography (CT), magnetic resonance imaging (MRI), X-rays, and ultrasound are listed in Table 3. Annual costs for the modalities were between €3.26 for MRI and €0.20 for ultrasound. The total annual cost for imaging was €6.51. Costs for mammography were not calculated, as these procedures were also performed within the framework of mammography screening in Germany.

**Table 3:** Costs for imaging procedures. Frequency represents the percentage of patients that utilized the modality; procedures represents the mean number of performed procedures

Modality	Frequency (%)	Procedures (*n*)	Annual costs per patient (€)
Computed tomography	22.4	0.39	2.45
Magnetic resonance imaging	17.1	0.31	3.26
X-ray	36.8	0.55	0.59
Ultrasound	47.4	0.19	0.20

#### Medicine procedures

In all, 28.9% of the patients underwent medical procedures such as acupuncture or local anesthesia (neural therapy) due to problems related to macromastia. The annual costs for medicine procedures were between €3.97 for acupuncture and €0.05 for chiropractic therapy. Total annual cost was €4.37 per patient. The frequency of procedures and the costs are listed in Table 4.

**Table 4:** Integrative medicine procedures: frequency and costs (Frequency represents the percentage of patients that utilized the modality; procedures represent the mean number of performed procedures.)

Modality	Frequency (%)	Procedures (*n*)	Annual costs per patient (€)
Acupuncture	11.8	0.67	3.97
Chiropractic therapy	2.7	0.09	0.05
Neural therapy	1.35	0.14	0.09
Dorn back therapy	1.35	0.14	0.24

#### Remedial procedures

Over half of the patients (57.9%) stated that they had been treated with physiotherapy to obtain relief from chronic pain and musculoskeletal problems resulting from macromastia. A mean of 8.37 visits per year had taken place before surgery, resulting in annual costs of €132.66. Over 60% of the patients had received massage treatment for pain relief, resulting in annual costs of €98.15. Costs for these and other remedial methods are listed in Table 5.

**Table 5:** Costs for remedial procedures

Modality	Patients (%)	Visits per year	Average duration (min)	Annual costs per patient (€)
Physiotherapy	57.9	8.37	16.78	132.66
Massage	61.8	8.52	17.89	98.15
Fango	31.6	4.69	4.63	104.21
Manual therapy	22.4	4.03	1.86	75.04
Osteopathy	15.8	0.83	5.17	54.78
Back therapy training	31.6	5.06	12.08	51.71
Nutritional courses	17.1	1.18	6.89	69.38
Electrotherapy	10.5	0.38	1.10	1.67
Thermal therapy	1.3		0.07	0
Heliotherapy	1.3	0.08	0.2	0.24

#### Rehabilitation

Only 5.3% of the patients reported that they had received orthopedic rehabilitation treatment before surgery, representing €17.86 per patient. Psychosomatic rehabilitation treatment was reported by 2.6%, representing costs of €323.40 per patient. Prevention treatment at a health resort or spa was used by 6.6% of the patients, with costs of €241.04 per patient (Table 6).

**Table 6:** Costs for rehabilitation procedures

Rehabilitation type	Patients (%)	Frequency	Annual costs per patient (€)
Orthopedic	5.3	0.15	75.31
Psychosomatic	2.6	0.05	64.68
Prevention treatment (health resort or spa)	6.6	0.08	90.39

#### Medication

A total of 172,949 days of medication were calculated, representing 2672.61 days of drug administration per patient. The total costs for medication for each patient were €882.35 for the period of time before reduction mammoplasty, or €74.64 per year and patient.

The highest costs for medication were caused by analgesics. The majority of the patients were taking analgesics due to pain from macromastia (67.1%). The total days of intake per patient between the start of symptoms and surgery were calculated as 1145.38 days. The total costs per patient were €276.67 for the period between the start of symptoms and surgery. The annual cost of analgesics per patient was €23.40.

Figures for antacid agents, antidepressant drugs/psychopharmaceuticals, relaxants, and ointments are listed in Table 7.

**Table 7:** Medication intake due to macromastia: frequency, duration, and costs

Type	%	Total days of intake	Days of intake per patient	Annual costs per patient (€)
Analgesics	67.1	79,031	1145.38	23.40
Antacid agents	22.4	12,994	341.95	8.42
Antidepressants/psychopharmaceuticals	11.8	17,460	239.18	8.41
Relaxants	15.8	5088	74.82	2.75
Ointment/dermatologic agents	27.6	44,558	665.04	10.50
Other	9.2	13,818	206.24	21.07
*Total*	–	17,2949	2672.61	74.64

#### Special brassiere

Special brassieres were needed by 51.3% of the patients for pain relief before reduction mammoplasty. On average, each patient had 3.12 special brassieres during the period before surgery. The costs were covered by insurance in only a single case. Costs for brassieres were around €70 per item, resulting in total preoperative costs of €218.40 per patient, or €18.47 per year.

#### Physical exercise

More than a third of the patients (34.2%) had attended exercise classes for a mean of 10.37 months before reduction mammoplasty. The average costs were €16.23 per patient and month, representing annual costs of €14.23. Among the patients, 18.4% had been members of a gym for a mean of 14.06 months before surgery, with mean costs of €11.00 per patient and month, representing annual costs of €13.08 per patient.

### Indirect costs

#### Travel expenses

As the questionnaire included questions about travel to and from various relevant activities such as consultations, nutritional courses, and physiotherapy, it was possible to calculate travel expenses. On average, each patient had traveled 130.93 km during the period before surgery. Assuming a cost of €0.30 per kilometer—the standard amount for commuter tax relief in Germany—this represents €3.32 per patient and year.

#### Indirect nonmedical costs: sick leave and employment

The main cost factor was loss of productivity due to sick leave resulting from symptoms of macromastia. Most of the patients were in employment before surgery (88.2%), with a mean of 28.73 work hours per week. One-third of the patients had a mean of 4.68 sick days per year before surgery. However, the symptoms had become worse over time and their ability to work had been restricted for a mean of 3.83 years before reduction mammoplasty. The total loss of GDP during the period from first symptoms to surgery was equivalent to €5555. Due to the variability of sick days per year in the preoperative period, the total costs for each patient had to be divided by the mean period of symptoms. The annual costs per patient were thus €470.

#### Implications

Each stakeholder in the health-care system has a different view of the costs. Society as a whole is the stakeholder with the highest total costs, due to the German health-insurance system and employee protection legislation. Costs for initiating objection proceedings against the health-insurance funds’ medical service departments if they declined to cover the costs for reduction mammoplasty were not included in this calculation, as the aim was to present a cost analysis of conservative treatment for macromastia.

#### Total costs per year and patient

**Fig. 2:**
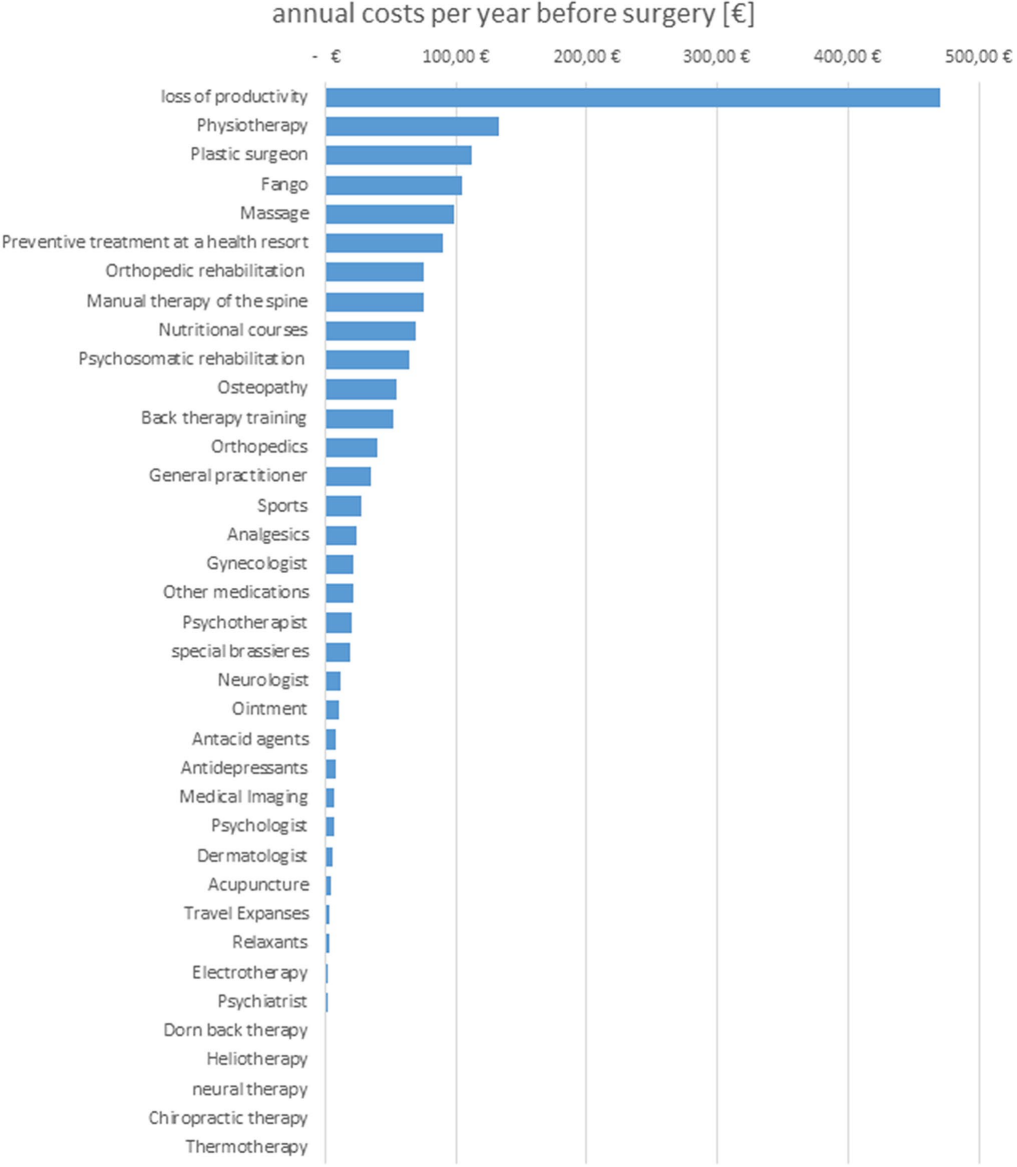
Proportion of costs per patient for treating the symptoms of macromastia on total annual costs of €1677 (€)

The total annual cost per patient amounted to €1677.55. Figure 2 shows the different annual costs in each category. The major proportion of the annual costs (28%) was due to loss of GDP resulting from sick leave, followed by costs for physiotherapy (8%) and plastic surgery consultations (7%). In total, medical procedures such as physiotherapy, massage, fango, back therapy training, nutritional courses, and manual therapy made up almost one-third of the costs (32%).Costs for medical consultation, medical treatment, physiotherapy, medication, rehabilitation, and loss of productivity are costs that have to be covered by society as a whole, due to the German health-care system. In the present cohort of patients with macromastia, these costs over the period of 11.82 years amounted to a total of €19,829.15 per patient.

#### Costs for insurance companies

Insurance companies and other cost-bearers cover the costs of medical consultations and imaging procedures. Acupuncture and chiropractic therapy also have to be covered by insurance. Costs for physical therapy, back therapy education, and nutritional education are subsidized at the level of €75 per course twice a year by the insurance funds. Rehabilitation measures and prevention treatments at health resorts, including travel expenses, are mainly covered by health insurance companies or pension insurance funds. Special brassieres were only covered by one health insurance company. Drug costs are covered by the health insurance companies, with the exception of the insurance excess amount that has to be paid by patients themselves.

A total of €1161.18 per year and patient have to be covered by insurance companies and other cost-bearers before reduction mammoplasty, representing €13,725 over the period of 11.82 years.

## Discussion

There have been several studies on the cost-effectiveness of surgical treatment for macromastia, but there is a lack of data on the complete costs of the condition. This questionnaire survey for the first time provided an opportunity to calculate the costs arising from conservative treatment for macromastia on the basis of real-world data. The limited data previously available are based on single cases and often do not include indirect and nonmedical costs—e.g., those due to loss of productivity [28]. Scholz et al. estimated the costs of conservative treatment for the 6 months before surgery as representing €4725 [28].

In contrast, the present study is based on real-world data, averaged for more than 70 patients with macromastia. The socioeconomic costs, including travel expenses and loss of productivity, are also taken into account. Although the costs calculated are lower than existing estimates, they appear to be reasonable. As the costs are averaged over a large cohort and a long period of time, their dynamic is flattened.

The annual costs for conservative treatment of macromastia are calculated on the basis of the data from the questionnaires completed by the patients. The median age for the start of symptoms was 26.48 years. The period from the beginning of symptoms to surgery was 11.82 years. Assuming hypothetically that all patients were to be treated conservatively without surgery until the end of their lives, with an average life expectancy of 82 years, the costs have to be multiplied. In this example, the total lifetime costs for conservative treatment for macromastia would be 55.52 × €1677.59 = €93,140 per patient.

However, it can be assumed that symptoms and problems due to macromastia will increase over time, leading to an increase in the annual costs, so that the total lifetime costs are likely to be even higher than calculated. On the other hand, costs due to loss of productivity will decrease after the age of 65–67 due to retirement.

Assuming that 1–5% of the female population suffer from macromastia [4], the costs of ineffective treatment can be regarded as high, quite apart from the pain and chronic problems of the women affected.

Decision-making on whether the costs of macromastia are to be covered by insurance is currently based on different criteria. It is mainly the nature of the surgery that is important whether it is only aesthetic or is medically indicated. Insurance companies often argue that reduction mammoplasty is a form of plastic surgery and decline to reimburse the costs. The evidence provided here of the total costs of macromastia may provide further arguments in support of insurance coverage.

Regardless of the costs for each category, the usefulness of each form of treatment has to be assessed. Several studies have evaluated the effect of weight reduction, for example, and found that it did not have any positive effects on the symptoms of macromastia [2, 11, 20]. The resources, including costs, that are devoted to weight reduction are therefore of very limited effect. There are no reports in the published literature describing any form of conservative treatment that leads to long-lasting reduction of the symptoms of macromastia.

### Limitations

In this study, costs were calculated from the start of symptoms to surgery with reduction mammoplasty, as the study cohort was recruited from this group of patients. There might, therefore, be some bias in the patient selection, since women with macromastia who do not have any symptoms will not seek medical treatment. It might be assumed that these patients do not need special treatment and so will not give rise to any additional costs. However, as one reasonable definition of macromastia makes the presence of chronic pain an obligatory element, the present cohort of patients is in fact representative of this diagnostic group.

Recall bias: As the patients were recruited several years after surgery, it is possible that the information which they provided about the years before surgery is not complete. Moreover, no exact information is available about variability in costs during the years before surgery. For example, patients might have consulted their family physician several times at the start of symptoms, before the diagnosis and the cause of pain were identified, with no further consultations being needed after the diagnosis. On the other hand, it is quite possible that the symptoms increase over time, with the frequency of appointments with a physiotherapist, for example, also increasing.

### Future prospects

Since it is known from several studies that conservative treatments such as weight reduction and exercise [11] fail to result in a reduced breast size and that physiotherapy, special brassieres, and medication do not have any long-lasting effects on symptom control [2, 11, 29] in patients with macromastia, the costs of conservative treatment could be saved if a causal therapy approach was to be pursued.

On the other hand, quality assessment is nowadays becoming increasingly important in the calculation of health-care costs, and the loss of quality of life also needs to be taken into account during therapeutic decision-making. A cost–utility calculation would, therefore, be helpful, and this should be the focus of further research.

## References

[1] Bässler R, ed. (1978) Pathologie der Brustdrüse. Spezielle pathologische Anatomie. ed. W Doerr, G Seifert, and E Uehlinger. 11 Springer-Verlag: Berlin. 283

[2] Gonzalez F (1993). Reduction mammaplasty improves symptoms of macromastia. Plast Reconstr Surg.

[3] Peters F (1991). Gutartige erkrankungen der brust: leitfaden für die praxis.

[4] Peters F (1998). Entwicklungsstörungen der mamma und deren behandlung. Der Gynäkologe.

[5] Peters F (1981). PRL, TSH, and thyroid hormones in benign breast diseases. Klinische Wochenschrift.

[6] Archer DF, Josimovich JB (1975). Response of serum prolactin to exogenous stimulation. Fertil Steril.

[7] Pega S (2006). Entwicklung eines neuen fragebogens zur qualitätssicherung der mammareduktionsplastik: der MRP-Bogen. Geburtshilfe Frauenheilkunde.

[8] Letterman G, Schurter M (1980). The effects of mammary hypertrophy on the skeletal system. Ann Plast Surg.

[9] Berg A, Stark B, Malec E (1994). Reduction mammaplasty: a way helping females with neck, shoulder and back pain symptoms. Eur J Plast Surg.

[10] Chadbourne EB (2001). Clinical outcomes in reduction mammaplasty: a systematic review and meta-analysis of published studies. Mayo Clin Proc.

[11] Collins ED (2002). The effectiveness of surgical and nonsurgical interventions in relieving the symptoms of macromastia. Plast Reconstr Surg.

[12] Glatt BS (1999). Retrospective study of changes in physical symptoms and body image after reduction mammaplasty. Plast Reconstr Surg.

[13] Horch RE, Jaeger K, Stark GB (1999). Lebensqualität nach Mammareduktionsplastiken. Handchir Mikrochir Plast Chir.

[14] Schnur PL (1997). Reduction mammaplasty: an outcome study. Plast Reconstr Surg.

[15] Shakespeare V, Cole RP (1997). Measuring patient-based outcomes in a plastic surgery service: breast reduction surgical patients. Br J Plast Surg.

[16] Sigurdson L (2007). Symptoms and related severity experienced by women with breast hypertrophy. Plast Reconstr Surg.

[17] Strittmatter HJ, Blecken SR, Neises M (2004). Lebensqualitätsverbesserung nach mammareduktionsplastik. Zentralblatt für Gynäkologie.

[18] Wagner DS, Alfonso DR (2005). The influence of obesity and volume of resection on success in reduction mammaplasty: an outcome study. Plast Reconstr Surg.

[19] Young, VL and ME Watson, eds. Breast reduction. Psychological aspects of reconstructive and cosmetic plastic surgery, ed. DB Sarwer, et al. 2006, Lippincott Williams and Wilkins: Philadelphia 189–207

[20] Zwiorek L (2011). Die Mammareduktionsplastik in Deutschland – Ergebnisse einer multizentrischen Erhebung. Geburtshilfe Frauenheilkd.

[21] Iwuagwu O (2006). Macromastia and carpal tunnel syndrome: is there an association?. Aesthetic Plast Surg.

[22] Pernia LR (2000). Carpal tunnel syndrome in women undergoing reduction mammaplasty. Plastic Reconstruct Surg.

[23] Hermans BJE (2005). Quality of life after breast reduction. Ann Plast Surg.

[24] Sood R (2003). Effects of reduction mammaplasty on pulmonary function and symptoms of macromastia. Plast Reconstr Surg.

[25] Klassen A (1996). Should breast reduction surgery be rationed? A comparison of the health status of patients before and after treatment: postal questionnaire survey. BMJ.

[26] Guthrie E (1998). Psychosocial status of women requesting breast reduction surgery as compared with a control group of large-breasted women. J Psychosom Res.

[27] Benditte-Klepetko H (2007). Hypertrophy of the breast: a problem of beauty or health?. J Women's Health.

[28] Scholz T (2008). Kostenanalyse der konservativen versus operativen therapie der makromastie. Handchir Mikrochir Plast Chir.

[29] American society of plastic surgeons. Evidence-based clinical practice guideline: reduction mammaplasty. 2011; [cited 07.07.2016]. Available from: https://www.plasticsurgery.org/Documents/Health-Policy/Guidelines/guideline-2011-reduction-mammaplasty.pdf. Accessed 07 July 2016

[30] Hermanns PM et al (2015) EBM 2015 - Kommentierter Einheitlicher Bewertungsmaßstab. Berlin, Springer Verlag. ISBN 978-3-662-46609-4, ISSN 2627-2636. 10.1007/978-3-662-46609-4

[31] Berufsverband Deutscher Osteopathischer Ärzteverbände (BDOÄ), Kosten einer osteopathischen Behandlung. [cited 27.12.2017]. Available from: https://bdoa.de/kosten_osteopathie.htm. Accessed 27 Dec 2017

[32] Bundesvertretung der Osteopathen in Deutschland (VOD e.V.). Wissenswertes über Osteopathie: Behandlung. [cited 27.12.2017]. Available from: https://www.osteopathie.de/osteopathie-behandlung. Accessed 27 Dec 2017

[33] Deutsche Rentenversicherung Bund. Reha-Bericht Update 2016. [cited 28.12.2017]; Available from: https://www.deutsche-rentenversicherung.de/SharedDocs/Downloads/DE/Statistiken-und-Berichte/Berichte/reha_bericht_update_2016.pdf;jsessionid=A6B2E8A847C54645D25263A0C25D6ACA.delivery1-8-replication?__blob=publicationFile&v=1

[34] Gesundheitsberichterstattung des Bundes. Leistungsfälle und Leistungstage sowie Tage je Fall von Rehabilitationsmaßnahmen der GKV-Versicherten. [cited 24.10.2017]; Available from: https://www.gbe-bund.de/gbe10/i?i=886:24793956D. Accessed 24 Oct 2017

[35] Gesundheitsberichterstattung des Bundes. Leistungstage und Leistungsfälle sowie Tage je Fall von Vorsorgekuren der GKV-Versicherten [cited 24.10.2017]; Available from: https://www.gbe-bund.de/gbe10/i?i=885:24793982D. Accessed 24 Oct 2017

[36] Statistisches Bundesamt. Bruttoinlandsprodukt (BIP) je Erwerbstätigen in Deutschland von 1991 bis 2017. [cited 9.4.2018]; Available from: https://de.statista.com/statistik/daten/studie/440518/umfrage/bruttoinlandsprodukt-je-erwerbstaetigen-in-deutschland/. Accessed 09 Apr 2018

